# Evolutionary mobility and genetic dynamics of MORFFO genes: shuttling among ancient plant lineages

**DOI:** 10.1111/nph.70986

**Published:** 2026-02-19

**Authors:** Paulo H. Labiak, Li‐Yaung Kuo, Blake D. Fauskee, Kenneth G. Karol

**Affiliations:** ^1^ Departamento de Botânica Universidade Federal do Paraná Caixa Postal 19031 81531‐980 Curitiba‐PR Brazil; ^2^ The New York Botanical Garden Center for Biodiversity and Evolution 10458 Bronx NY USA; ^3^ Institute of Molecular and Cellular Biology National Tsing Hua University 30013 Hsinchu Taiwan; ^4^ Department of Biology Duke University Durham 27708 NC USA

**Keywords:** anemia, chloroplast genome, ferns, HGT, mobile DNA, Schizaeales

## Abstract

- Plastid genomes (plastomes) of land plants are characterized by their architectural and genic content stability. However, fern plastomes exhibit unexpected dynamism, characterized by the presence of mobile protein‐coding genes (CDS) – Mobile Open Reading Frames in Fern Organelles (MORFFOs).
- We investigate the evolutionary dynamics of MORFFOs in 30 species of Anemiaceae (Schizaeales), an ancient lineage of ferns, focusing on their transposition, substitution patterns, codon usages, and RNA editing patterns.
- MORFFOs expand plastome size and occur in diverse intergenic regions, exhibiting dynamic locations, genealogies, and exceptionally high substitution rates compared with canonical plastid CDS. Sliding window and codon usage analyses demonstrate that MORFFOs are under purifying selection but exhibit distinct codon preferences that deviate from those of other plastid CDS, suggesting functional constraints. Phylogenetic incongruence between MORFFOs and other plastid CDS, along with their extraordinary substitution rates and mobility, implies their replication outside plastids.
- Our findings highlight that MORFFOs are dynamic, potentially selfish genetic elements capable of transcription, translation, and replication independently from plastomes, and fern plastomes might acquire these mobile CDS through frequent horizontal gene transfer and possibly intracellular gene transfer.

## Resúmen

- Los genomas de los plastidios (plastomas) de las plantas terrestres se caracterizan por la estabilidad de su arquitectura y de su contenido génico. Sin embargo, los plastomas de los helechos muestran un dinamismo inesperado, caracterizado por la presencia de genes móviles que codifican proteínas (MORFFOs, por sus siglas en inglés).
- Nosotros investigamos la dinámica evolutiva de los MORFFOs en 30 especies de Anemia (Anemiaceae), un linaje antiguo de helechos. Centrándonos en su transposición, patrones de sustitución, uso de codones y patrones de edición de ARN.
- Los MORFFOs aumentan el tamaño del plastoma y se presentan en diversas regiones intergénicas, exhibiendo ubicaciones dinámicas, genealogías y tasas de sustitución excepcionalmente altas en comparación con los genes codificantes de proteínas (CDS) plastídicos canónicos. Los análisis de ‘sliding windows’ y de uso de codones demuestran que los MORFFOs están bajo selección purificadora, pero presentan preferencias de codones distintas, que se desvían de las de otros CDS plastídicos, lo que sugiere restricciones funcionales. La incongruencia filogenética entre los MORFFOs y otros CDS plastídicos, junto con sus extraordinarias tasas de sustitución y movilidad, sugiere su replicación fuera de los plastidios.
- Nuestros resultados destacan que los MORFFOs son elementos genéticos dinámicos, potencialmente egoístas, capaces de transcripción, traducción y replicación de forma independiente a los plastomas; además, los plastomas de los helechos podrían adquirir estos CDS móviles a través de transferencias horizontales de genes (HGT) frecuentes y, posiblemente, transferencias intracelulares de genes (IGT).

## Introduction

Plastid genomes (plastomes) in land plants are typically characterized by a highly conserved organization and genic content, in contrast to their pre‐endosymbiotic cyanobacterial ancestors, which exhibit dynamic genomic architecture and frequently engage in horizontal gene transfer (HGT), as is common among prokaryotes (Gross *et al*., 2008; Wicke *et al*., 2011). Most land plant plastomes have a quadripartite structure composed of two inverted repeats (IRs) flanking a large single‐copy (LSC) and a small single‐copy (SSC) region, and encode a core set of *c*. 80–87 protein‐coding genes (CDS) within a relatively compact genome of *c*. 120–160 kbp (Wicke *et al*., 2011; Gitzendanner *et al*., 2018). Large‐scale rearrangements, such as inversions or translocations, are rare, contributing to the general conservation of gene order (synteny) across plastomes (Wicke *et al*., 2011). However, size variation has been identified in plastomes of a few land plant lineages due to changes in organization and genic content. Significant size reductions in plastomes are observed in some groups, such as the gymnosperms (Cronn *et al*., 2008; McCoy *et al*., 2008), Cactaceae (Sanderson *et al*., 2015), Convolvulaceae (Funk *et al*., 2007), Orchidaceae (Yang *et al*., 2013), and ferns (Schizaeales and *Stromatopteris* Mett. in Gleicheniales; Labiak & Karol, 2017; Du *et al*., 2022; Ke *et al*., 2022). In many such cases, the entire suite of *ndh* genes (*c*. 10 kbp) has been lost. Conversely, when observed, plastome expansions are frequently attributable to IR expansions that duplicate adjacent genes (Li *et al*., 2016; Kim *et al*., 2020; Du *et al*., 2022; Jiang *et al*., 2024). By contrast, the acquisition of foreign genes and thus enriching genic content via HGT or intracellular gene transfer (IGT) into plastomes is exceedingly rare and has been reported in a few groups of angiosperms to date (Straub *et al*., 2013; Filip & Skuza, 2021; Jo *et al*., 2024; Cauz‐Santos, 2025). One such case is from the parasitic plant *Rafflesia* R. Br. (Rafflesiaceae), which possesses plastome‐like ‘vestiges’ containing plastid genes derived from its host (Molina *et al*., 2014). Although progress has been made in acknowledging these putative intergenomic transfers, the physical integration of these transferred elements remains unresolved.

Plastomes of ferns have generally mirrored the conserved features of those in other land plant lineages (Du *et al*., 2019, 2022), with only a few cases exhibiting dramatic size variation that can largely be explained by IR expansion or gene loss (Labiak & Karol, 2017; Kuo *et al*., 2018; Du *et al*., 2019, 2022). However, recent discoveries have revealed novel, lineage‐specific plastid CDSs in ferns (Robison *et al*., 2018; Song *et al*., 2018), which challenge the paradigm of plastome conservatism. Notably, some of these sequences, termed Mobile Open Reading Frames (ORF) in Fern Organelles (MORFFOs), appear to be predominantly found in ferns and are absent in nearly every other land plant lineage (Kim & Kim, 2018, 2020; Robison *et al*., 2018; Lehtonen & Cárdenas, 2019; Kim *et al*., 2023; Kuo *et al*., 2024). MORFFOs are particularly intriguing due to their widespread occurrence across fern lineages, their uncertain evolutionary origin, and their unusual genomic behavior (Robison *et al*., 2018; Kuo *et al*., 2024). Unlike canonical plastid genes, MORFFOs are highly variable in presence and genomic location, often forming large clusters up to 11 kbp and contributing to notable increases in plastome size (Robison *et al*., 2018; Lehtonen & Cárdenas, 2019). To date, MORFFOs have been reported in plastomes from nearly half of all fern families (Lehtonen & Cárdenas, 2019; Kuo *et al*., 2024). In the family Pteridaceae, three distinct MORFFO genes (*morffo1*–*morffo3*) have been identified in various locations, including intergenic regions within the LSC and the *rrn16–rps12* intergenic region in the IR (Robison *et al*., 2018). In Ophioglossaceae, *morffo1* and *morffo2* occur in both plastomes and mitogenomes (Kim & Kim, 2018; Kuo *et al*., 2024). Additionally, MORFFO‐like sequences have been found in some fern nuclear genomes and several organellar genomes in other land plant lineages (Kuo *et al*., 2024). Their sporadic distribution and lack of clear orthologs outside ferns suggest possible origins via HGT and/or IGT (Kim & Kim, 2018; Robison *et al*., 2018; Kuo *et al*., 2024). To date, however, no CDSs have been confirmed as resulting from HGT from a plastome to another plastome in land plants (Cauz‐Santos, 2025), rendering MORFFOs potentially unprecedented in their diversity and evolutionary origin, despite their prevalence in ferns.

Within ferns, the order Schizaeales stands out for its remarkable variation in plastome organization, which is complicated by IR expansions and the loss of the entire *ndh* suite of genes. Diverging more than 200 Ma (Schuettpelz & Pryer, 2009; Testo & Sundue, 2016; Nitta *et al*., 2022), Schizaeales includes families that differ markedly in plastome architecture and genic content. Schizaeaceae, for instance, exhibits extreme plastome reduction and IR reconfiguration (Labiak & Karol, 2017; Ke *et al*., 2022). Plastomes of *Actinostachys* Wall. (Schizaeaceae) rank among the smallest known in ferns and exhibit the complete loss of all *ndh* and *chl* genes, likely reflecting a mycoheterotrophic gametophytic phase (Labiak & Karol, 2017; Ke *et al*., 2022). By contrast, Anemiaceae – the sister family to Schizaeaceae – maintains relatively conserved plastome organization and gene content, albeit with moderate expansions primarily due to the insertion of MORFFO‐like sequences into intergenic regions. Recent sequencing efforts have revealed that such insertions are present in *c*. 80% of all studied Anemiaceae species, suggesting that MORFFO integration is a recurring feature in the family. Given the contrasting plastomic features between Anemiaceae and its sister lineage, Schizaeaceae, this system provides a unique opportunity to investigate the evolutionary origins and behavior of MORFFOs. In particular, Anemiaceae plastomes offer insights into the dynamics of these unusual sequences and their role in plastome evolution.

In this study, we conducted comprehensive analyses of Anemiaceae plastomes to not only reconstruct the plastome phylogeny of this group but also investigate the diversity, distribution, and evolutionary patterns of MORFFOs. We began by conducting phyloplastomic sampling across this fern family. By analyzing these plastomes, we characterized their organization and genic content, including MORFFO insertion sites. We then reconstructed a robust plastome phylogeny to trace the evolutionary histories of MORFFOs and assess their substitution rates, codon usage, sequence divergence, and RNA editing patterns relative to canonical plastid genes. Based on this integrated evidence, we tested the hypothesis that MORFFOs originated through HGT and/or IGT and demonstrate that their mobility has played a significant role in the evolution of fern plastomes.

## Materials and Methods

### DNA extraction, genome sequencing, assembly, and annotation

Samples included fresh‐collected, silica‐dried leaves, and herbarium specimens. Total genomic DNA was extracted using the Qiagen DNeasy Plant Mini Kit (Valencia, CA, USA) following the manufacturer's protocol. For most samples, TrueSeq libraries were prepared and sequenced using the Illumina HiSeq 2500 at Cold Spring Harbor Laboratories. The sample of *Anemia phyllitidis* (L.) Sw. (collection no. *Kuo 4249*) was sequenced by Novogene Co., Ltd (Beijing, China) on the Illumina NovaSeq X Plus platform. The resulting paired‐end reads were processed using Geneious Prime (Kearse *et al*., 2012; v.2020.2.4, Biomatters Ltd, Auckland, New Zealand). For most species, end regions with >5% chance of error per base were trimmed, and the resulting reads were assembled *de novo* in Geneious Prime. For *Anemia phyllitidis*, specifically, plastome assembly was carried out using the *de novo* approach as implemented in NOVOPlasty v.4.3.3 (Dierckxsens *et al*., 2017), with the *rbcL* sequence from a different *A. phyllitidis* voucher as reference. The draft plastome assembly was iteratively polished using Pilon v.1.24 (Walker *et al*., 2014). Initial plastome annotations were performed in Geneious Prime using published plastome annotations of related taxa. Gene content, protein translation, putative start and stop codons, and introns were verified using the find ORFs tool as implemented in Geneious Prime. Additionally, tRNA genes were annotated using tRNAscan‐SE 2.0 (Chan *et al*., 2021). Putative RNA editing events necessary for protein translation were predicted by visual inspection of ORFs and with the R script ReFernment (Robison & Wolf, 2019). Plastome structure, gene content, and general characteristics were then compared to selected fern plastomes available on NCBI (http://www.ncbi.nlm.nih.gov/).

### Plastid RNA editing analysis

For *A. phyllitidis*, transcriptomic data were generated from the same specimen (*Kuo 4249*) used for genomic data and plastome assemblies. RNA was extracted from flash‐frozen green sporophyte tissue using the E.Z.N.A. Plant RNA Kit from Omega Bio‐Tek (R6827; Omega Bio‐Tek, Norcross, GA, USA), with additional treatment using DNase I (69 182; Millipore Sigma, Darmstadt, Germany) to reduce genomic DNA contamination. RNA concentration was quantified using a Qubit 2 Flurometer (Thermo Fisher Scientific Inc., Walden, MA, USA) with the Qubit RNA High Sensitivity Quantification Assay kit (Q32852; Thermo Fisher Inc.). A cDNA library was constructed using the NEBNext Ultra II Library Prep (E7775; New England Biosciences, Ipswich, MA, USA) with ribosomal depletion probes designed for plant samples supplied by New England Biosciences as part of a beta test agreement.

Plastid RNA editing sites for *A. phyllitidis* were identified by extracting all CDS and MORFFO sequences using Geneious Prime, including 100 bp of flanking sequence upstream and downstream of each gene. These sequences were concatenated into a single multi‐FASTA file. The RNA editing detection pipeline from Fauskee *et al*. (2025) was implemented to detect potential RNA editing sites. In this pipeline, RNA reads were trimmed twice with Trimmomatic (v.0.39), first in paired‐end mode using the following settings: LEADING:3 TRAILING:3 SLIDINGWINDOW:4:15 MINLEN:36 to remove adapters and low‐quality reads, then in single‐end mode using the HEADCROP:13 setting, which removes the first 13 bases in each read in which GC (guanine‐cytosine) content was nonuniform. RNA reads were then mapped to the plastid CDS for each species using Bowtie2 v.2.2.4 (Langmead & Salzberg, 2012), and a BAM file was generated using Samtools v.1.14 (Li *et al*., 2009). The total number of RNA reads mapped to each site in each gene, as well as the number of reads with each nucleotide present at that site, were calculated using bam‐readcount v.0.8.0 (Khanna *et al*., 2022) and custom Linux commands. This ultimately outputs a TSV file showing the base present in the DNA sequence at each site for each analyzed gene, the total number of mapped RNA reads at each site, and the number of mapped reads for each of the four nucleotides.

Putative RNA editing sites were identified and characterized using the R pipeline from Fauskee *et al*. (2025). A site was classified as edited if ≥ 10 RNA reads mapped to the site, and ≥ three reads and ≥ 10% of those mapped reads showed the edited nucleotide. (e.g. a T mapped to a C for a C‐to‐U editing site). Manual inspection was performed in uncommon regions with very low RNA read coverage. The pipeline also characterized the amino acid change induced by each RNA editing event, the codon position at which it occurs, the efficiency of each editing site, and several other features. RNA editing efficiency is defined as the proportion of mapped RNA reads displaying the edited base (e.g. the number of reads with a T mapped to a C divided by the total number of reads mapped to that site).

### Plastome sampling

*Anemia* is the only extant genus in Anemiaceae, with *c*. 115 species (Mickel, 2016). The ingroup included newly generated plastomes for 30 species of *Anemia*: *Anemia adiantifolia* (L.) Sw., *A. colimensis* Mickel, *A. collina* Raddi, *A. delicatula* Mickel, *A. dregeana* Kunze, *A. elegans* (Gardner) C. Presl, *A. gardneri* Hook., *A. glareosa* Gardner, *A. hirsuta* (L.) Sw., *A. hispida* Kunze, *A. irwinii* Mickel, *A. labiakii* Mickel, *A. lanipes* C. Chr., *A. mandiocana* Raddi, *A. marginalis* (Sav.) Christenh., *A. marginata* Mickel, *A. mexicana* Klotzsch, *A. millefolia* Gardner, *A. mohriana* Christenh., *A. multiplex* Mickel, *A. oblongifolia* (Cav.) Sw., *A. phyllitidis* (L.) Sw., *A. retroflexa* Brade, *A. rotundifolia* Schrad., *A. rutifolia* Mart., *A. salvadorensis* Mickel & Seiler, *A. simii* Tardieu, *A. tomentosa* (Sav.) Sw., *A. warmingii* Prantl, and A. *wightiana* Gardner, plus a previously published plastome of *A. adiantifolia* (Du *et al*., 2022). These species were chosen to represent the main clades within *Anemia*, as recovered in previous phylogenies (Labiak *et al*., 2015). For the other members of the Schizaeales, 11 species of Schizaeaceae were included (*Actinostachys digitata* (L.) Wall., *A. pennula* Hook., *Microschizaea fistulosa* (Labill.) C.F. Reed, *M. tenella* (Kaulf.) C.F. Reed, *Schizaea dichotoma* (L.) Sm., *S. elegans* (Vahl) Sw., *S. medusa* L.Y. Kuo, B.F. Ke, Fay W. Li & Rouhan, *S. pectinata* (L.) Sw., *S. poeppigiana* J.W. Sturm, *S. pusilla* Pursh, and *S. sprucei* Hook.) (Labiak & Karol, 2017; Ke *et al*., 2022), and five accessions of *Lygodium –* three from prior studies (two specimens of *L. japonicum* (Thunb.) Sw., and *L. microphyllum* (Cav.) R. Br.) (Gao *et al*., 2013; Kim *et al*., 2014) and two newly sequenced for this study (*L. palmatum* (Bernh.) Sw. and *L. reticulatum* Schkuhr). GenBank accession numbers are provided in Supporting Information Dataset S1.

### Phylogenomic analyses of plastomes

CDSs were extracted for 86 plastid‐encoded genes across all taxa. Each gene was individually aligned using the ‘translation alignment’ tool implemented in Geneious Prime. Gene alignments with pairwise identity < 70% were excluded due to homology uncertainty (*rpl21*, *rpl23*, *rps15*, *ycf2*, *rpoC2*, *ndhF*, *cemA*, *matK*, and *ycf1*). The resulting alignments were visually inspected, and regions of uncertain homology were trimmed using Gblocks 0.91.1 (Castresana, 2000; Talavera & Castresana, 2007) in codon mode with a relaxed set of parameters (b2 = 3, b5 = half). Maximum likelihood (ML) phylograms were generated using IQ‐TREE v.2 (Minh *et al*., 2020). Three codon partitioning schemes were analyzed: all codon positions (‘ALL’), first+second positions (‘codon12’), and third positions only (‘codon3’). Model selection was performed using ModelFinder (Kalyaanamoorthy *et al*., 2017) with the Bayesian Information Criterion with the setting of ‘‐m TESTNEWMERGE’ and ‘‐rcluster 100’. Support values were calculated using 1000 ultrafast bootstrap replicates (Hoang *et al*., 2018).

### Identification of MORFFOs

MORFFOs were initially detected using the Geneious Prime ‘find ORFs’ tool with a minimum ORF length of 500 bp. Similar positions were annotated sequentially (e.g. *morffo1*, *morffo2*, *morffo3*). BLASTn searches were conducted using previously described MORFFOs from Robison *et al*. (2018) and Lehtonen & Cárdenas (2019) to identify homologous sequences in Anemiaceae. For species lacking large insertions, whole plastome and BLASTn comparisons were used to identify potential homologs. tBLASTn was used to search for MORFFO‐like sequences in other fern plastomes and *Anemia* transcriptomes. Protein sequences of MORFFOs identified in *Anemia* were queried against FTOL v.1.6 (Nitta *et al*., 2022), the poly‐A enriched transcriptomes of *A. phyllitidis* (Qi *et al*., 2018) and *A. tomentosa* (One Thousand Plant Transcriptomes Initiative, 2019), and the ribosomal depletion transcriptome of *A. phyllitidis* by this study, which was assembled using Trinity (Grabherr *et al*., 2011). The MORFFOs were extracted and aligned using a codon‐aware approach with MACSE v.2 (Ranwez *et al*., 2011), which also identified frameshift mutations in the coding sequences. The MORFFOs were then reannotated following the alignment results.

### Substitution patterns of MORFFOs

To further assess whether *Anemia* MORFFOs behave like CDS, substitution patterns were analyzed using a sliding window approach. Specifically, pseudogene‐like sequences detected with frameshift mutations by MACSE were excluded, and stop codons were removed from the alignments. Because some *Anemia morffo2* lacked a stop codon, the genic boundary between *morffo2* and the 3′‐adjacent *morffo1* remains ambiguous. Therefore, we also analyzed nucleotide substitution patterns across the *morffo2‐morffo1* intergenic spacer (IGS), hereafter referred to as ‘*morffo1 + 2*.’ With these alignments, preliminary ML phylograms of *Anemia* MORFFOs were generated using the same IQ‐TREE parameters mentioned earlier. A sliding window analysis (window size = 90 bp; step size = 3 bp) was implemented, and pairwise DNA sequence divergences (pi) for different codon positions were generated using a customized Python script with the Biopython library (Cock *et al*., 2009). Ratios of nonsynonymous to synonymous substitutions (dN/dS) were estimated by HyPhy (Kosakovsky *et al*., 2020) under the MG94 codon substitution model (Muse & Gaut, 1994) using the corresponding preliminary ML phylograms.

### MORFFOs phylogeny

To investigate evolutionary relationships of *Anemia* MORFFOs with expanded sampling, we incorporated the *morffo1* and *morffo2* alignments from Kuo *et al*. (2024) (22 additional fern families). The *morffo1 + 2* alignment was split based on IGS boundaries identified through substitution patterns (see earlier in ‘Substitution patterns of MORFFOs’ in the Materials and Methods section). The *morffo3* alignment was expanded using sequences from Robison *et al*. (2018) and newly identified sequences from our BLAST searches (see the ‘Identification of MORFFOs’ in the Materials and Methods section). All alignments were refined using MACSE. Model selection and codon partitioning were performed with ModelFinder (BIC, recluster = 100). ML gene phylograms were generated in IQ‐TREE v.2 with 1000 ultrafast bootstrap replicates.

### Relative synonymous codon usage

Relative synonymous codon usage (RSCU) for *Anemia* MORFFOs and 86 plastid‐encoded CDSs was calculated using the cusp package in EMBOSS (Rice *et al*., 2000). We further compared the RSCU patterns of MORFFOs in *A. phyllitidis* with those of its nuclear, plastid, and mitochondrial CDSs. To retrieve nuclear CDSs, we identified Benchmarking Universal Single‐Copy Orthologs (BUSCO) genes from the newly generated transcriptome (see earlier ‘Identification of MORFFOs’ in the Materials and Methods section) using BUSCO v.6 and the Viridiplantae ODB10 dataset (Tegenfeldt *et al*., 2025). To obtain mitochondrial CDSs, we incorporated the workflow of Cárdenas & Lehtonen (2023) and Kuo *et al*. (2024). First, HybPiper v.2 (Johnson *et al*., 2016) was used to assemble mitochondrial gene CDSs, using *A. phyllitidis* genome‐skimming DNA reads and fern mitochondrial exon sequences by Cárdenas & Lehtonen (2023) as our input and blastx reference, respectively. The resulting hybpiper output was then used as seeds for mitogenome assembling in NOVOPlasty. Finally, mitochondrial CDSs were annotated and manually verified in Geneious Prime. In this RSCU comparison, only CDSs longer than 500 bp were analyzed to avoid stochastic biases resulting from short gene length. The amino acids methionine (M) and tryptophan (W) were excluded from the analysis because only a single codon encodes each. Principal component analysis (PCA) was used to assess differences in RSCU results.

### Substitution rate estimation

Relative and absolute substitution rates were calculated for each MORFFO and plastid‐encoded CDS using tip‐to‐root distances in their single‐gene phylograms, normalized by those from the plastome phylogram and chronogram, respectively. Specifically, the 86‐CDS ML phylogram constructed from the matrix including all sites (see ‘Phylogenomic analyses of plastomes’ in the Materials and Methods section) was used as our plastome phylogram. Using this phylogram, the corresponding plastome chronogram was generated using treePL (Smith & O'Meara, 2012). Calibration points included a crown group age maximum of 231.11 mya for Schizaeaceae+Anemiaceae (Testo & Sundue, 2016; Nitta *et al*., 2022), and a minimum crown node age of 136 mya for Anemiaceae based on *Pelletixia* and *Ruffordia* fossils from the Early Cretaceous (Skog, 1982; Dettmann & Clifford, 1992).

All single‐gene phylograms for all CDSs were reconstructed using IQ‐TREE v.2. All 86 plastid‐encoded CDS single‐gene phylograms were inferred under a topological constraint from the plastome ML phylogram. Model selection and codon partitioning followed the same IQ‐TREE settings described earlier (see ‘MORFFOs phylogeny’ in the Materials and Methods section). Previously reconstructed MORFFO ML phylograms were used (see ‘MORFFOs phylogeny’ in the Materials and Methods section) after removing *Anemia simii* (due to horizontal transfer likely from other fern lineages; see the Results section). To obtain conservative rate estimates that are presumed to remain under functional constraints, we also performed analyses excluding pseudogene‐like MORFFOs (i.e. sequences containing frameshift mutations) by removing their tips from the MORFFO ML phylograms. To calculate relative substitution rates, all non‐Anemiaceae tips from all phylograms were also removed, and subphylograms were generated from every internode of the trimmed single‐gene phylograms. Tip‐to‐root branch lengths for each subphylogram were summed, and these values were divided by the corresponding sums derived from the plastome subphylogram. Absolute substitution rates were calculated similarly, using the corresponding branch length sums from the plastome subchronograms as the denominators. To ensure consistent ‘roots’ (i.e. placement of the most recent common ancestor) across comparisons, we included only those single‐gene subphylograms in which all descendant taxa had formed a monophyletic group in both the plastome phylogram and chronogram. All comparisons and calculations were performed using a custom R script with the phytools (Revell, 2012), ape (Paradis *et al*., 2004), adephylo (Jombart *et al*., 2010), and phangorn (Schliep, 2011) packages.

## Results

### Plastome architecture and identification of MORFFO

**Fig. 1:**
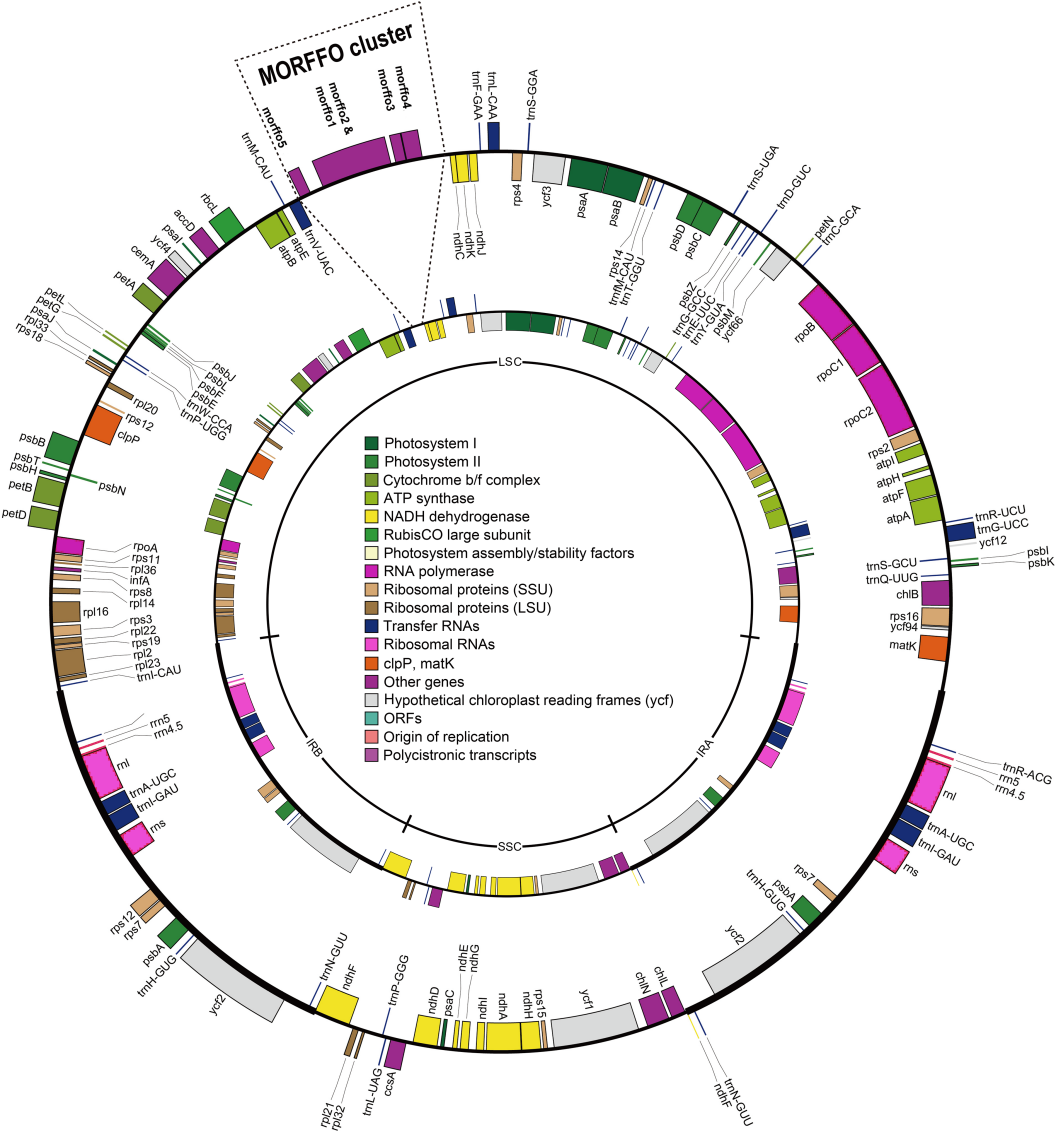
Plastomes of Anemiaceae with (*Anemia hirsuta*, outer plastome) or without (*A. labiakii*, inner plastome) a large insertion between the *trnV‐UGC* and *ndhC* genes, where the Mobile Open Reading Frames in Fern Organelles (MORFFO) cluster is located.

We assembled 29 complete Anemiaceae and two Lygodiaceae plastomes in this study. The only incomplete plastome was *Anemia wigthiana*, which was recovered in two contigs separated by an ambiguous assembly in the LSC. All plastomes have a quadripartite organization composed of one LSC, SSC, and two IRs. Gene content and architecture are relatively stable among the different lineages. The plastomes of *Anemia* are composed of 117 genes, 86 CDSs, four rRNAs, and 27 tRNAs. Plastome length varied from 158,086 bp (in *A. marginalis*) to 169,465 bp (in *A. elegans*). A graphical representation of two *Anemia* plastomes is depicted in Fig. 1. The two plastomes of *Lygodium* are also similar in gene content and architecture, with 86 CDS, four rRNAs, and 27 tRNAs. The total lengths of each plastome and each partition (LSC, SSC, and IR) are summarized in Table 1.

**Table 1:** Features of the *Anemia* plastomes.

Species	Plastome size (bp)	GC%	LSC (bp)	SSC (bp)	IR (bp)	MORFFO (location)
*Anemia adiantifolia*	162 393	39.6	89 081	22 636	25 338	
*Anemia colimensis*	166 588	40.7	92 673	22 481	25 717	2, 3 (*petE‐petL* IGS)
*Anemia collina*	168 038	38.4	94 285	22 773	25 490	2, 3, 4, 5 (*ndhC‐trnV* IGS)
*Anemia delicatula*	166 735	38.3	94 573	22 522	24 820	1, 2, 3, 4, 5 (*ndhC‐trnV* IGS)
*Anemia dregeana*	162 446	38.0	88 894	22 764	25 394	4 (*ndhC‐trnV* IGS)
*Anemia elegans*	169 497	38.1	87 094	22 419	29 992	2, 3, 4 (*ycf2‐trnN* IGS)
*Anemia gardneri*	165 424	37.7	91 317	22 811	25 648	1, 2, 5 (*ndhC‐trnV* IGS)
*Anemia glareosa*	168 948	38.3	94 994	22 506	25 724	1, 2, 4 (*ndhC‐trnV* IGS)
*Anemia hirsuta*	168 525	38.3	94 941	22 778	25 403	1, 2, 3, 4, 5 (*ndhC‐trnV* IGS)
*Anemia hispida*	167 933	38.4	94 304	22 791	25 419	1, 2, 3, 4, 5 (*ndhC‐trnV* IGS)
*Anemia irwinii*	165 681	38.3	92 612	22 263	25 403	1, 2, 4, 5 (*ndhC‐trnV* IGS)
*Anemia labiakii*	161 880	37.9	87 874	22 564	25 721	
*Anemia lanipes*	166 839	38.3	89 086	22 695	27 529	2 (*ycf2‐trnN* IGS)
*Anemia mandiocana*	168 634	38.7	94 907	22 705	25 511	1, 2, 3, 4 (*ndhC‐trnV* IGS)
*Anemia marginalis*	158 086	38.5	87 751	22 657	23 839	
*Anemia marginata*	167 176	38.2	94 896	22 548	24 866	1, 2, 3, 4, 5 (*ndhC‐trnV* IGS)
*Anemia mexicana*	164 632	39.8	90 676	22 590	25 683	
*Anemia millefolia*	161 184	37.7	87 954	22 500	25 365	4 (*ndhC‐trnV* IGS)
*Anemia mohriana*	163 391	39.0	91 911	22 790	24 345	2 (*ndhC‐trnV* IGS)
*Anemia multiplex*	162 142	37.9	88 565	22 789	25 394	3, 4 (*ndhC‐trnV* IGS)
*Anemia oblongifolia*	167 429	38.3	93 997	22 718	25 357	1, 2, 4, 5 (*ndhC‐trnV* IGS)
*Anemia phyllitidis*	161 330	38.0	87 555	22 769	25 503	2, 3, 4 (*ndhC‐trnV* IGS)
*Anemia retroflexa*	168 383	38.4	94 501	22 566	25 658	1, 2, 3, 4 (*ndhC‐trnV* IGS)
*Anemia rotundifolia*	166 859	38.4	93 396	22 625	25 419	2, 3 (*ndhC‐trnV* IGS)
*Anemia rutifolia*	166 856	38.3	94 647	22 507	24 851	1, 2, 3, 4, 5 (*ndhC‐trnV* IGS)
*Anemia salvadorensis*	161 286	37.8	87 652	22 774	25 430	
*Anemia simii*	167 595	38.3	93 835	22 514	25 623	1, 2, 3 (*ndhC‐trnV* IGS)
*Anemia tomentosa*	168 532	38.2	94 744	22 432	25 678	1, 2, 3, 4 (*ndhC‐trnV* IGS)
*Anemia warmingii*	163 652	38.2	90 357	22 775	25 260	1, 2, 5 (*ndhC‐trnV* IGS)
*Anemia wightiana*	166 643	38.3	92 762	22 585	25 648	1, 2, 4, 5 (*ndhC‐trnV* IGS)

IR, inverted repeat; LSC, large single copy; MORFFO, Mobile Open Reading Frames in Fern Organelles; SSC, small single copy.

Except for MORRFOs, gene content and order were identical among *Anemia* plastomes. The largest plastomes generally contained several insertions, ranging from 2400–9400 bp. These insertions included MORFFOs that were typically found in gene clusters and located between *ndhC* and *trnV‐UAC* in the LSC region in most species. Exceptions include MORFFOs located between *ycf2* and *trnN‐GUU* in the IR of *A. elegans* and *A. lanipes*, as well as MORFFOs located between *psbE* and *petL* in the LSC of *A. colimensis* (Table 1). In most instances, these MORFFOs shared the same start codons.

**Fig. 2:**
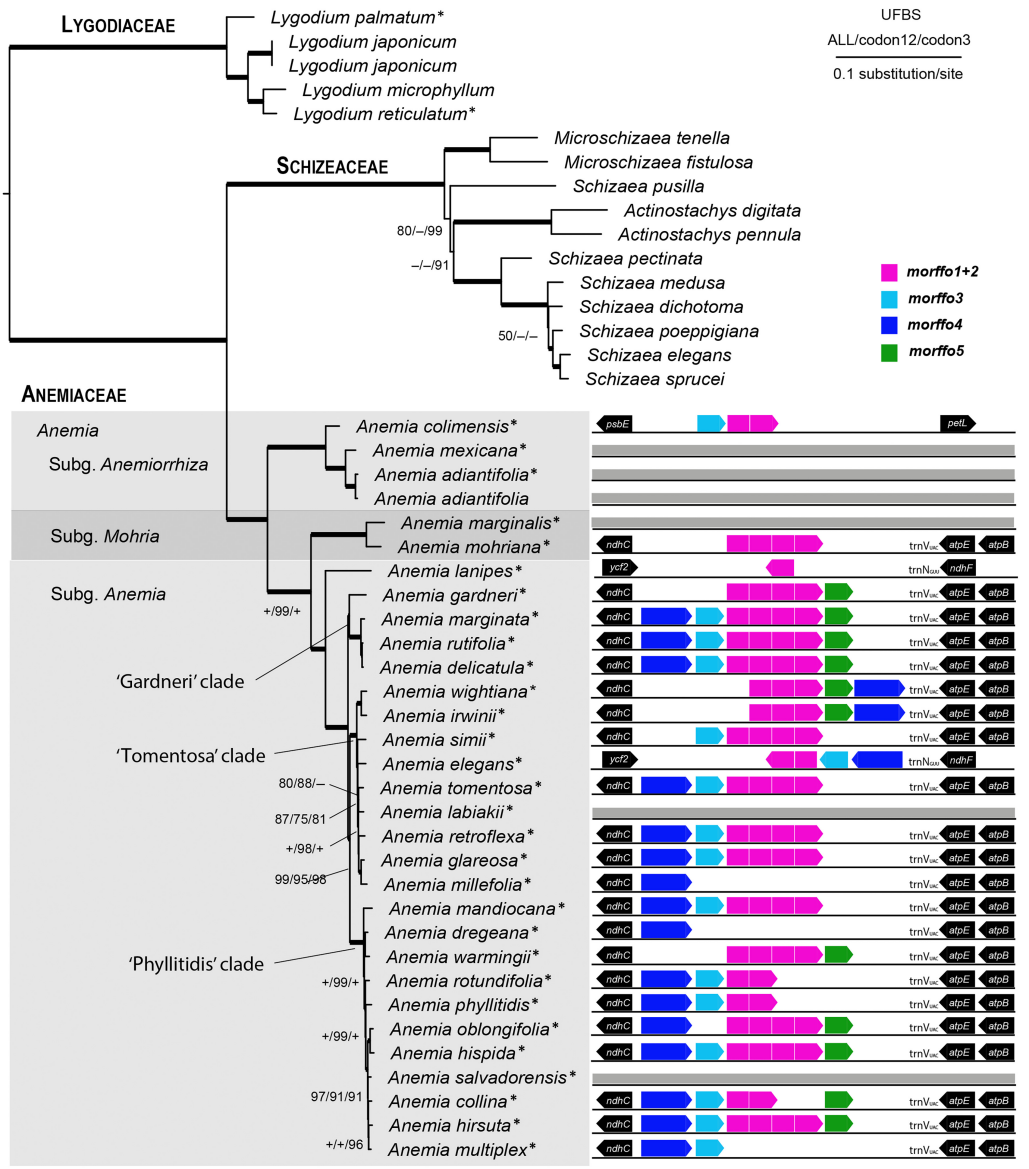
Phylogram of the Schizaeales based on 86 plastid protein‐coding genes (CDS). The main clades recognized by Labiak *et al*. (2015) are indicated at the left. The presence and position of each Mobile Open Reading Frames in Fern Organelles (MORFFO) are indicated at the right. Values shown along branches are maximum likelihood ultrafast bootstrap support (UFBS) for the three partitions used in our analyses; ‘+’ and ‘–’ indicate values of 100 and 50 or less, respectively. Branches with UFBS values of 100 are shown in bold. Asterisks indicate the newly sequenced plastomes.

We found at least five MORFFOs in *Anemia*. Three correspond to *morffo1*, *morffo2*, and *morffo3*, as described by Robison *et al*. (2018), based on our BLAST similarity searches (Dataset S1). Because these MORFFOs (including their IGS; Table 1) were present in 20 of 30 species (66.6%) and could be easily aligned, they were treated as homologous and named accordingly. However, these three MORFFOs were present only with partial sequences in some species, such as *A. dregeana*, *A. elegans*, *A. gardneri*, *A. irwinii*, *A. lanipes*, *A. marginata*, *A. warmigii*, *and A. wightiana* (Fig. 2). Notably, these MORFFO sequences were sometimes found as a single and large ORF (*c*. 4500 bp) or with internal stop codons that split the large ORF into up to four smaller ORFs of different lengths. *Morffo4* and *morffo5*, on the other hand, seem to be exclusive to Schizaeales, sharing only a slight resemblance with MORFFOs from other fern plastomes. *Morffo4* and *morffo5* were present in 19 of 30 (63.3%) *Anemia* species (Table 1) but only in species belonging to the three derived clades.

MORFFO*s* were not found in *Anemia mexicana*, *A. adiantifolia*, *A. labiakii*, *A. marginalis*, *A. millefolia*, or *A. salvadorensis* plastomes. Consistent with this finding, the intergenic regions between *ndhC* and *trnV‐UAC* in these species were significantly smaller, ranging from 718 bp to 917 bp. Although MORFFOs were not as evident as in most species, a few smaller ORFs (< 300 bp) found in *A. gardneri*, *A. millefolia*, and *A. phyllitidis* were somewhat similar to MORFFOs identified in other species. *Morffo4* and *morffo5* were also identified in other Schizaeales plastomes. Outside of Schizaeales, however, only a few tBLASTn hits met the cutoff criteria – covering at least one‐third of the query length and with sequence identity above 30% (Dataset S1). Under these criteria, *morffo4* was identified with weak similarity (30%) in the plastomes of *Mankyua chejuense* (GenBank accession no.: KP205433) and *Pyrrosia petiolosa* (GenBank accession no.: MT210541).

Sequence‐similar copies of MORFFOs were not detected in two *Anemia* polyA‐enriched transcriptomes (*A. phyllitidis* and *A. tomentosa*). Notably, highly similar copies (similarity >90%) were detected in the ribosomal‐depleted transcriptome for *A. phyllitidis* (Dataset S1); however, these sequences differed from those found in the *A. phyllitidis* plastome. In addition, no RNA editing was detected in the plastid‐encoded MORFFOs of *A. phyllitidis*, in contrast to the majority of other plastid‐encoded CDSs (Dataset S2). Although five MORFFO sites showed editing frequencies above 10% with more than two edited reads, our manual inspection of the read mapping profile confirmed that these signals originated from reads derived from other genes, likely other nonplastid‐encoded MORFFO copies.

### Phylogenomic analyses

The final nucleotide dataset comprised 47 taxa and 86 CDS, totaling 72 105 aligned nucleotides. The ML phylogenomic analyses (Figs 2, S1) recovered the main clades within Schizaeales, representing the three families traditionally recognized: Anemiaceae, Lygodiaceae, and Schizaeaceae.

In Anemiaceae, the results corroborate, in most respects, those presented by Labiak *et al*. (2015), offering better resolution for poorly resolved clades in that study. Subgen. *Anemiorrhiza* was identified as the first divergent lineage (ultrafast bootstrap support (UFBS) = 100), followed by subgen. *Mohria* (UFBS = 100) (Fig. 2). Within the subgen. *Anemia*, *Anemia lanipes* resolved as the first divergent lineage (UFBS = 100), and the remaining members were composed of three derived clades, including ‘gardneri’, ‘tomentosa’, and ‘phyllitidis’ (Fig. 2).

### Substitution patterns and rates of MORFFOs

**Fig. 3:**
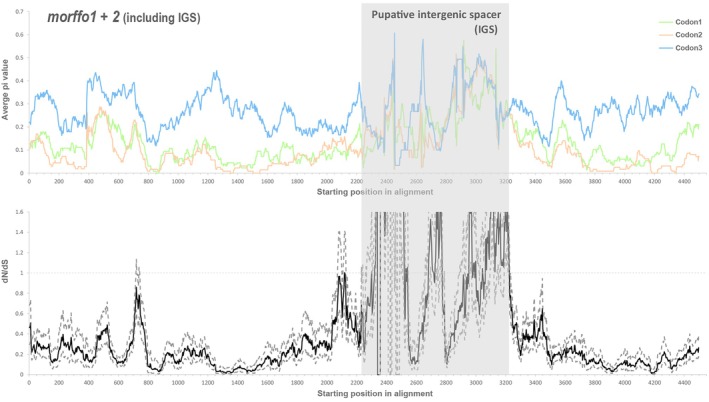
Sliding window analysis of *Anemia morffo1 + 2* (two MORFFO genes and their intergenic spacer) with a window size of 90 bp and step size of 3 bp. The upper panel shows the average pairwise nucleotide diversity (pi) across different codon positions. The lower panel displays dN/dS ratios, with the solid line representing the mean and dashed lines indicating the 95% confidence intervals.

**Fig. 4:**
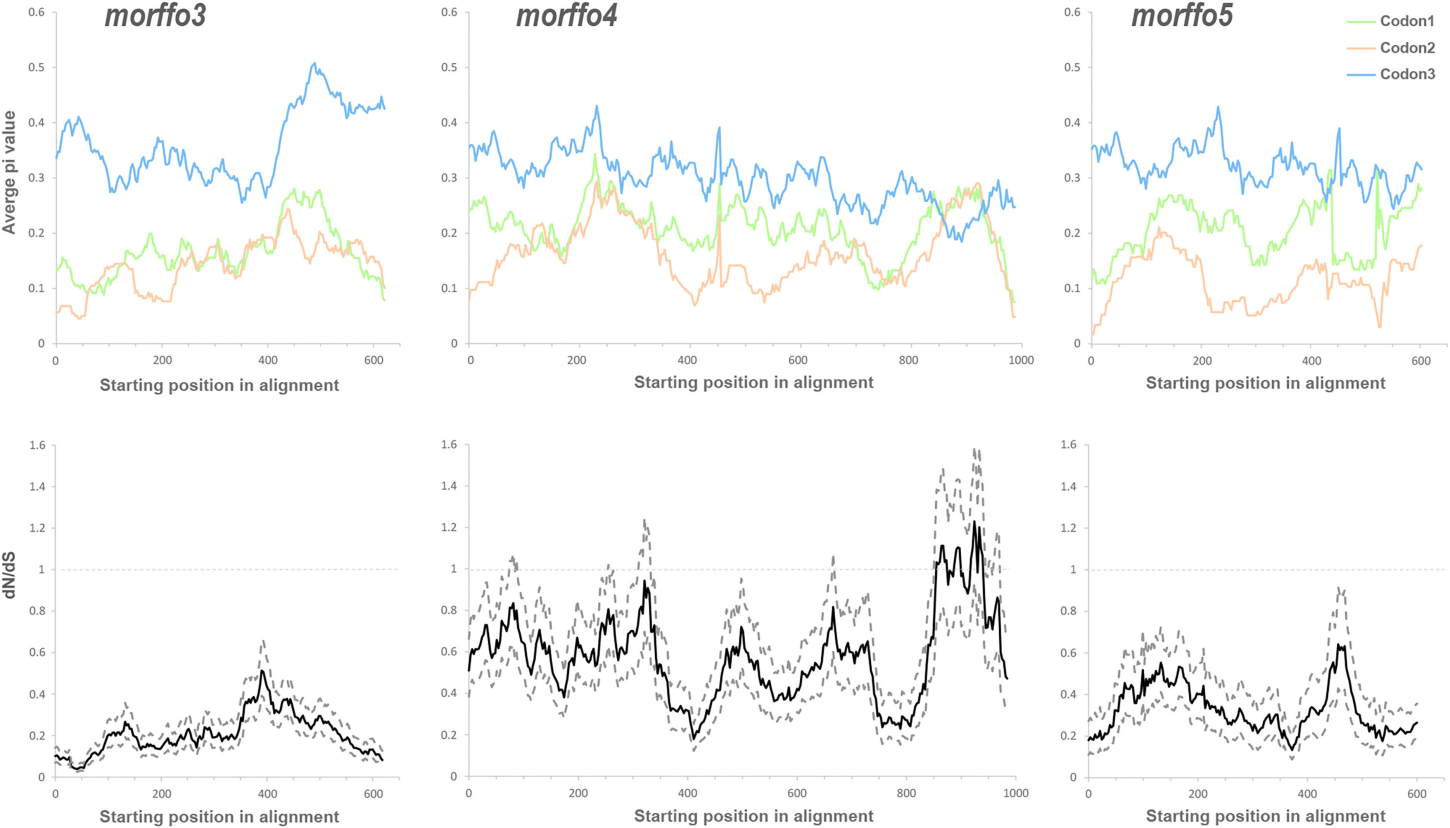
Sliding window analysis of other *Anemia* Mobile Open Reading Frames in Fern Organelles (MORFFOs) with a window size of 90 bp and step size of 3 bp. The upper panel shows the average pairwise nucleotide diversity (pi) across different codon positions. The lower panel displays the ratios of nonsynonymous to synonymous substitutions (dN/dS), with the solid line representing the mean and dashed lines indicating the 95% confidence intervals.

**Fig. 5:**
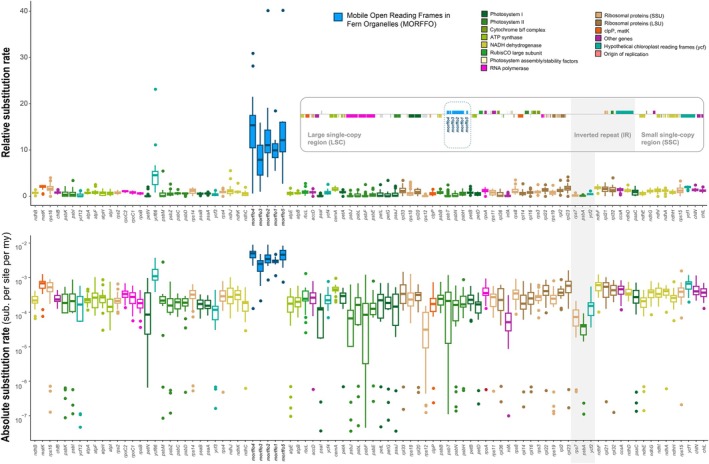
Relative (upper) and absolute (lower) substitution rates of nonpseudogenized Mobile Open Reading Frames in Fern Organelles (MORFFOs) and 86 plastid protein‐coding genes in *Anemia*. The boxplots show the 25^th^–75^th^ percentiles (boxes), median values (horizontal lines within the boxes), whiskers representing 1.5× the interquertile range, and points beyond these rangers as outliers.

The sliding window analyses further revealed selection constraints across the entire MORFFO gene family (Figs 3 and 4). Overall, their dN/dS ratios were lower than 1, with substitutions being more conserved in the first two codon positions compared to the third (Figs 3 and 4). In *morffo1 + 2*, we also identified a region with elevated dN/dS and substitution rates across all three putative codon positions (Fig. 3), which is presumably the noncoding IGS between *morffo1* and *morffo2*. Figs 5 and S2 present relative and absolute substitution rates of *Anemia* MORFFOs alongside those of the canonical plastid CDS. These MORFFOs exhibited exceptionally high rates, *c*. 10 to 15 times higher than other plastid‐encoded CDSs. As expected, MORFFO substitution rates were higher when pseudogenes were included (Fig. S2) than when they were excluded (Fig. 5). Another notable pattern is that CDSs in the IRs showed lower rates than those outside of the IRs (Figs 5, S2).

### Relative synonymous codon usage (RSCU)

**Fig. 6:**
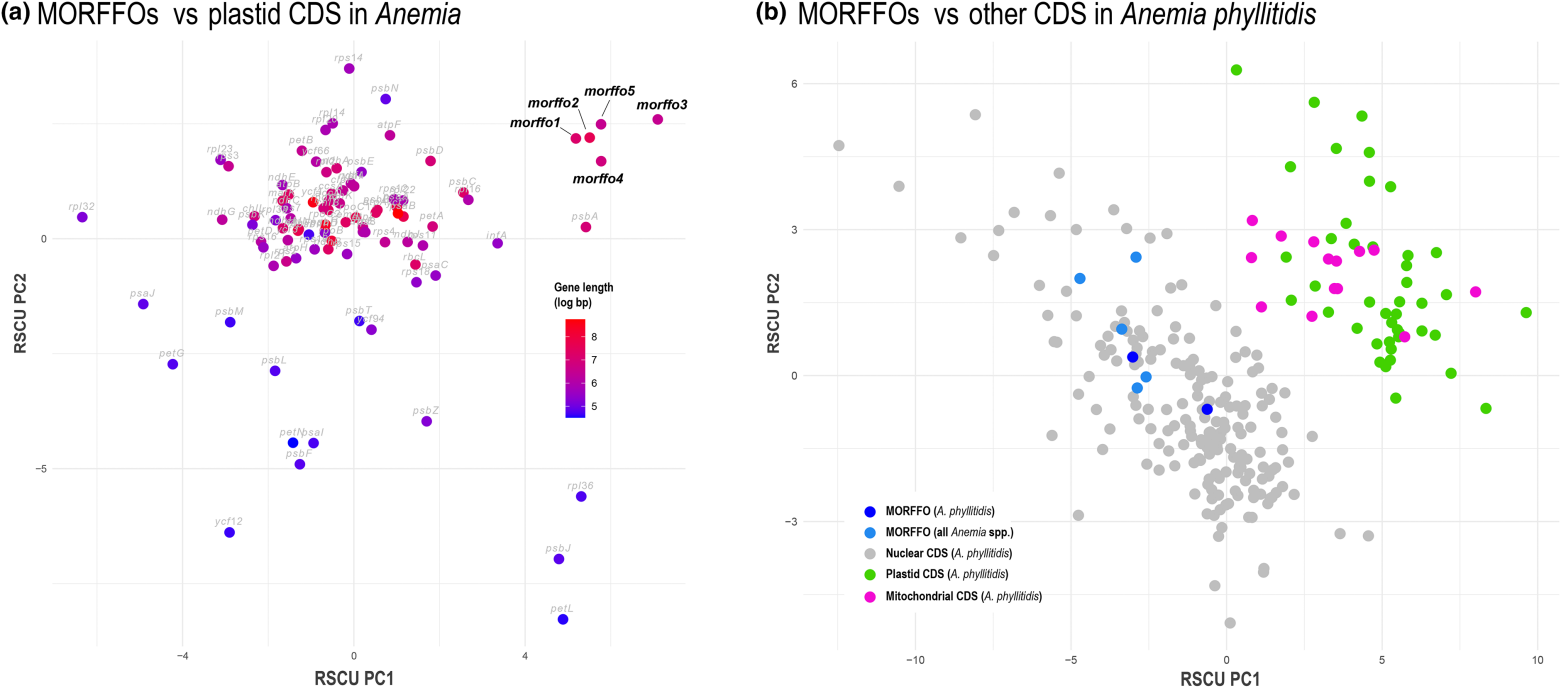
Relative synonymous codon usage (RSCU) principal component analysis plots of four Mobile Open Reading Frames in Fern Organelles (MORFFOs) and 86 plastid protein‐coding genes (CDSs) in *Anemia* (a), and MORFFOs, nuclear, plastid, and mitochondrial CDSs in *A. phyllitidis* (b).

Fig. 6a illustrates the PCA dimensions of RSCU of 86 CDS and four MORFFOs from *Anemia* plastomes. As expected under genetic drift, the genes with shorter lengths (< 150 bp) tended to be fixed and exhibited more biased codon usage, and thus were found more scattered in the plot (Fig. 6a). Except for MORFFOs, plastid‐encoded genes of substantial lengths displayed similar codon usage patterns, clustering closely together (Fig. 6a). By contrast, MORFFOs showed distinct codon usage patterns compared with other plastid‐encoded genes and formed a separate cluster further shows that the RSCU profiles of *A. phyllitidis* MORFFOs cluster with nuclear BUSCO CDSs (totally 185 complete single‐copy genes), and are well‐separated from both plastid (*n* = 44) and mitochondrial CDSs (*n* = 14).

### MORFFO phylogeny

**Fig. 7:**
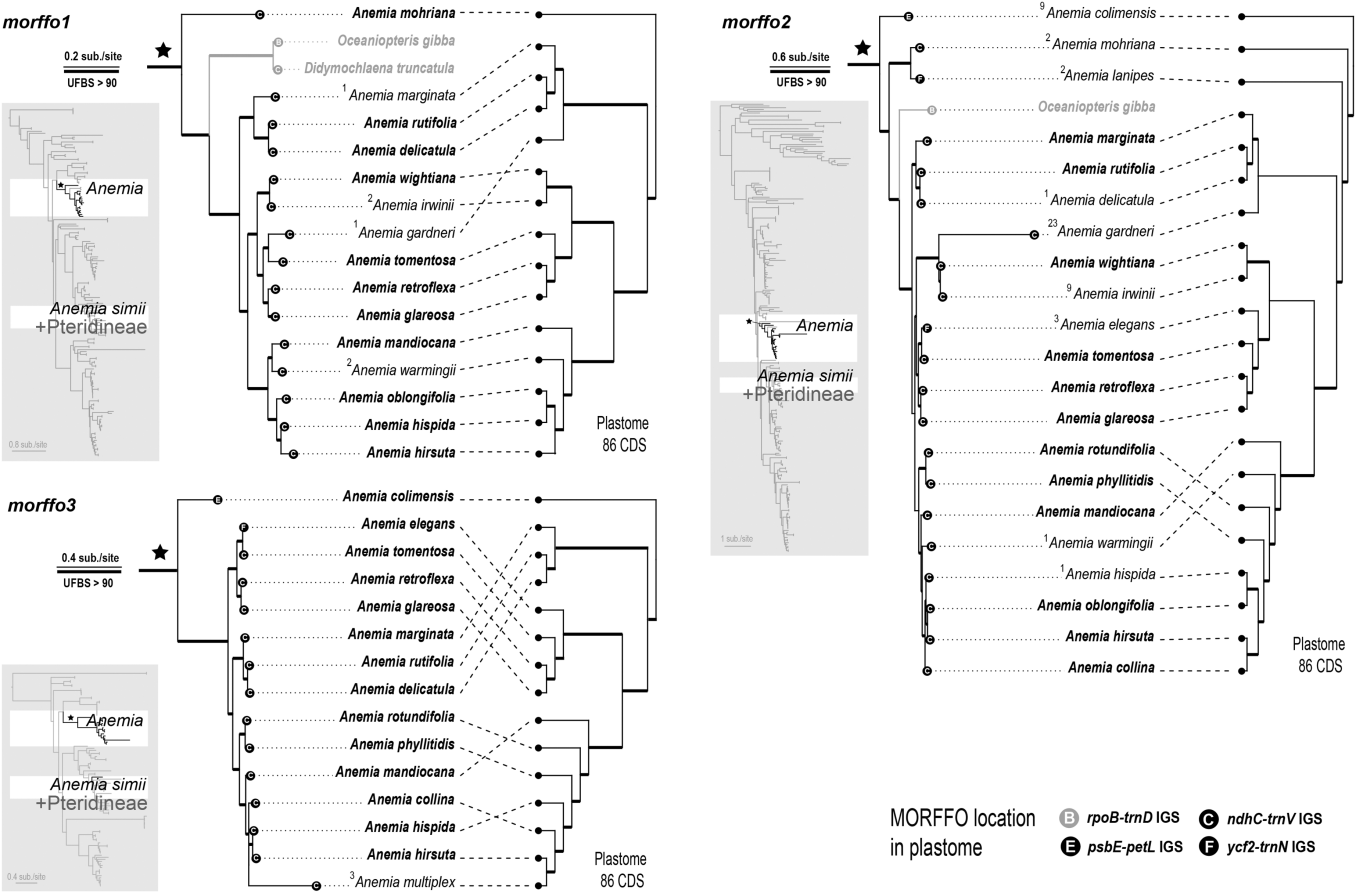
Phylogenies of *morffo1*, *morffo2*, and *morffo3* found in *Anemia* (left) aligned with the plastome topology (right). Numbers shown along branches are maximum likelihood ultrafast bootstrap support (UFBS) values. Branches with UFBS values of 90 are shown in bold. The values on the taxon names indicate the number of frameshift mutations found in Mobile Open Reading Frames in Fern Organelles (MORFFO) sequences. The left and lower panels show their full phylograms highlighted with *Anemia* MORFFO sequences.

**Fig. 8:**
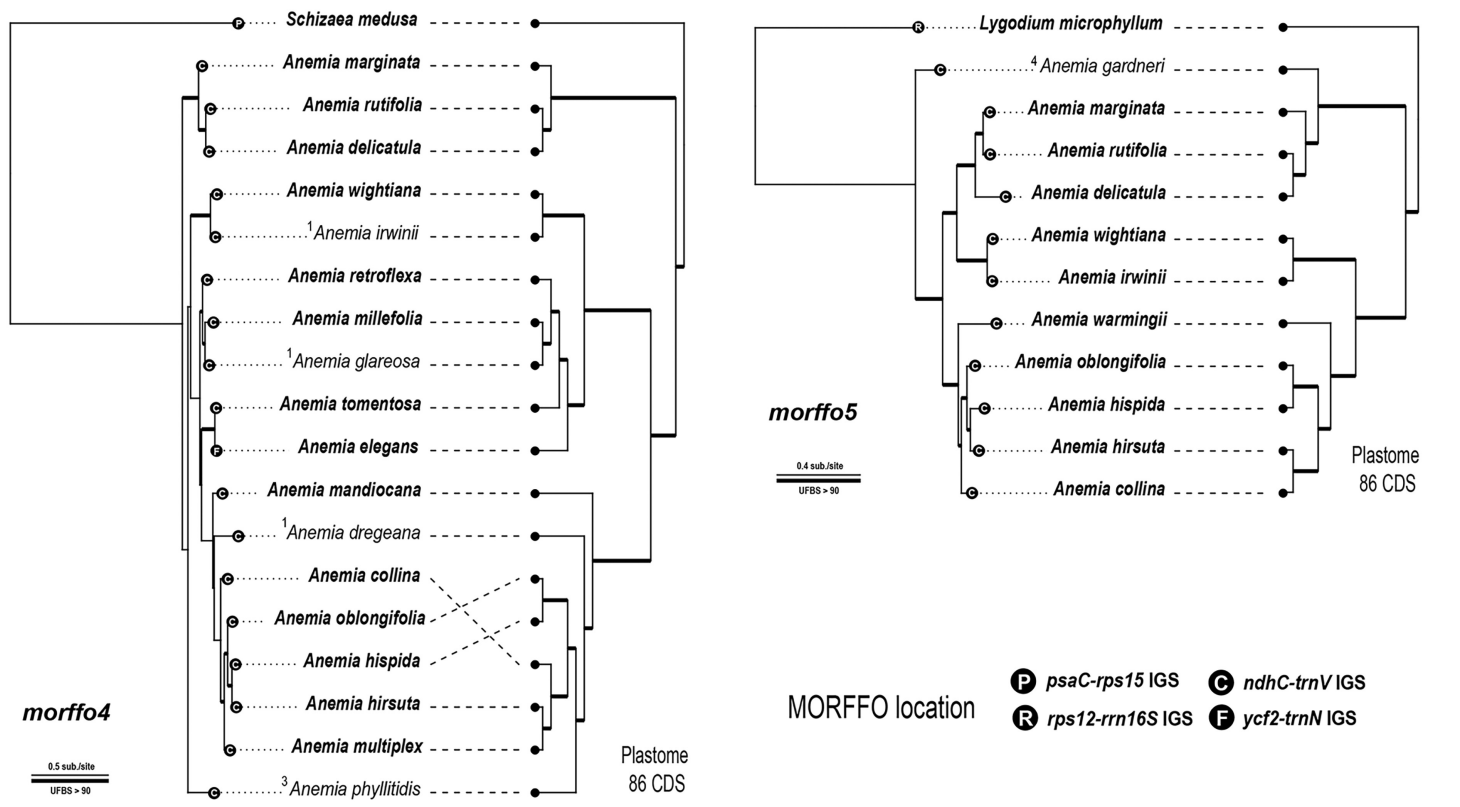
Phylogenies of *morffo4* and *morffo5* found in *Anemia* (left), aligned with the plastome topology (right). Numbers shown along branches are Maximum likelihood ultrafast bootstrap support (UFBS) values. Branches with UFBS values of 90 are shown in bold. The values on the taxon names indicate the number of frameshift mutations found in Mobile Open Reading Frames in Fern Organelles (MORFFO) sequences.

The phylograms of *Anemia* MORFFOs are shown in Figs 7 and 8 and S3–S5. All MORFFOs exhibited genealogies incongruent with the plastome phylogeny. *Morffo1*, *morffo2*, and *morffo3* phylogenies revealed that *Anemia* sequences were distantly related to other Schizaeales sequences and were not monophyletic (Figs 7, S3–S5). Notably, *A. simii* MORFFOs were phylogenetically distant from other *Anemia* sequences, and, instead, nested in those derived from different fern lineages, predominantly from Pteridineae (Polypodiales) ferns (Fig. 7). MORFFOs from other fern lineages were found nested within *Anemia* MORFFOs, including *morffo1* and *morffo2* of *Oceaniopteris gibba* (Labill.) Gasper & Salino (Aspleniineae; Polypodiales), and *morffo1* of *Didymochlaena truncatula* (Sw.) J. Sm. (Polypodiineae; Polypodiales) (Fig. 7). MORFFO gene phylogenies of *Anemia* species only partially align with the plastome phylogeny and strong conflict (e.g. UFBS > 90) within *Anemia* can also be easily found (Figs 7 and 8). In some cases, the terminal branches of *Anemia* MORFFO phylogenies were exceptionally long, likely due to gene pseudogenization, as indicated by frameshift mutations in the MACSE alignments.

## Discussion

### Phylogenomic topology with improved resolution

The phylogenomic results presented here (Fig. 2) support most of the relationships recovered in previous studies for the Schizaeales (Skog *et al*., 2002; Wikström *et al*., 2002; Labiak *et al*., 2015; Labiak & Karol, 2017; Ke *et al*., 2022). These include the recognition of the three main family‐level lineages: Anemiaceae, Lygodiaceae, and Schizaeaceae. Generic circumscriptions are also consistent with what is currently accepted, except for one example in the Schizaeaceae. Ke *et al*. (2022) proposed recognizing three genera in this family: *Actinostachys*, *Microschizaea*, and *Schizaea sensu stricto*. These genera were supported by phylogenomic analysis, except for the position of *S. pusilla*, which was recovered as sister to either *Schizaea* or *Schizaea + Actinostachys*, depending on the data matrices, models, and partitions used. The results presented here support the latter scenario, albeit with reduced support (Fig. 2).

For Anemiaceae, in particular, our phylogenetic analysis corroborates many of the previously recovered relationships for the backbone of Anemiaceae (Labiak *et al*., 2015). Generally, the three main clades, which correspond to the subgenera *Anemia*, *Anemiorrhiza*, and *Mohria*, were recovered here with high UFBS (Fig. 2). Within the *Anemia s.s*. clade (subgenus *Anemia*), we obtained a better‐resolved phylogeny (Figs 2, S1) than that of Labiak *et al*. (2015). In the current analysis, the first divergent lineage is *A. lanipes*, a species endemic to Madagascar. This relationship was also recovered in Labiak *et al*. (2015), and it is corroborated in our phylogenomic analysis. In our analysis, *A. gardneri* was recovered as sister to the *A. rutifolia* clade (*A. delicatula*, *A. marginata*, *and A. rutifolia*). This contrasts with the results of Labiak *et al*. (2015), in which *A. gardneri* was recovered as sister to the remaining clades of *Anemia*, namely, the phyllitidis and tomentosae clades. It should be noted, however, that the relationships recovered for these clades by Labiak *et al*. (2015) had low support values in both the Bayesian and ML analyses, which may be due to the limited amount of data used in that study.

Our results also supported the monophyly of the ‘tomentosa’ and the ‘phyllitidis’ clades of Labiak *et al*. (2015). In the ‘tomentosa’ clade, the polytomy among the *Anemia elegans*, *A. flexuosa*, and the *A. simii* clades was better resolved, but still without full support in our analysis. A similar pattern was recovered within the ‘phyllitidis’ clade, in which the relationships were better resolved but still without full support (Fig. 2). At the base of this clade, the relationships of *A. mandiocana* and *A. rotundifolia*, which were recovered as a polytomy in Labiak *et al*. (2015), were resolved as a grade that includes *A. mandiocana* as sister to the rest, followed by a clade with *A. dregenea*, *A. rotundifolia*, and *A. warmingii*. The remaining relationships were the same as those in Labiak *et al*. (2015), except for the close relationship between *A. hirsuta* and *A. multiplex*, which were then recovered in different clades (Fig. 2).

Noteworthy is that the Indian endemic *Anemia wightiana* was recovered as sister to the Brazilian endemic *A. irwinii* (Fig. 2). This sister relationship was unexpected – although possible – because it would require a long‐distance dispersal event between the two continents. However, our current sampling limits our ability to draw a more precise conclusion, making it necessary to include additional African and Malagasy samples for more robust biogeographical analyses.

### Signature of MORFFOs as atypical protein‐coding genes in plastomes

MORFFOs in fern plastomes were first identified by their recognizable ORFs, with similar amino sequences across fern taxa. Their expression and function as CDS, however, remain largely unexplored. Due to the absence of a conserved stop codon position, genic boundaries between some MORFFOs are also unclear, as showcased by *morffo1 + 2* in *Anemia* (Fig. 3). To address this issue, we attempted to detect MORFFO expression in transcriptomes of two *Anemia* species. Additionally, our empirical evidence from the sliding window analyses confirmed that substitution patterns of these *Anemia* MORFFOs resemble those of CDS with functional constraint (Figs 3 and 4). These MORFFOs are subject to not only translational selection with constrained codon usage (Fig. 6) but also to purifying selection, as indicated by dN/dS ratios that are predominantly below one across entire genes (Figs 3 and 4). This purifying selection is particularly evident at the first two codon positions (Figs 3 and 4), in which nucleotide substitutions often result in changes in amino acids. By contrast, selection at the third codon position is much more relaxed, following a pattern typical of coding genes (Figs 3 and 4). These results also allowed for a better delimitation of the noncoding IGS between *morffo1* and *morffo 2* (Fig. 3).

Despite exhibiting signatures typical of CDS under purifying selection, we found that MORFFOs have usually frequent pseudogenization (detailed in the ‘Dynamism and mobility of MORFFOs in plastomes’ in the Discussion section) and other atypical signatures compared to other plastid‐encoded genes. First, MORFFOs are often oriented opposite to the transcriptional direction of flanking operons, and/or located far from operon promoters that are conserved in land plants, including ferns (Shiina *et al*., 2005; Shahar *et al*., 2019). *Anemia* MORFFOs in this study are good examples: They are mostly inserted within the *trnV–ndhC* IGS, inside the *atpB* operon (Ghulam *et al*., 2012; Shahar *et al*., 2019), yet they are inverted relative to the transcriptional direction of the nested operon (Figs 2 and 5). Similar arrangements have also been observed in other MORFFOs (Lehtonen & Cárdenas, 2019; Kim *et al*., 2023; Kuo *et al*., 2024). Second, although these MORFFOs are transcribed, they apparently undergo no RNA editing (Dataset S2), unlike the majority of plastid‐encoded CDS in most ferns, in which post‐transcriptional C‐to‐U and U‐to‐C edits are frequent (Fauskee *et al*., 2025). For other *Anemia* plastid‐encoded CDSs, RNA editing has been found in 69 (80%) of these genes, with an overall occurring frequency of 0.55% across all sites (Dataset S2). However, none were detected in *A. phyllitidis* MORFFOs, which comprise 2490 bases. Lastly, their codon usage patterns differ significantly from those of canonical plastid CDS. As shown in the PCA plots of their RSCU, MORFFOs form a separate cluster from other plastid and also mitochondrial genes (Fig. 6), nested within the nuclear gene cluster (Fig. 6b). These differences in codon usage bias may reflect selection pressures associated with diverse translational ‘habitats’ represented by different genomic counterparts in plant cells. Taken together, MORFFOs appear to have transcription and translation decoupled from other plastid‐encoded CDS, are unlikely to use the plastid genetic machinery, and are very possibly expressed outside of the plastid, potentially within the nucleus.

### Dynamism and mobility of MORFFOs in plastomes

Since structural rearrangements and recombination events are infrequent in plastomes, the synteny of plastid genes is well‐preserved (Du *et al*., 2022; Cauz‐Santos, 2025), as also observed in the ancient fern lineage *Anemia* (Fig. 1). Due to this linkage, plastid genes are usually stably positioned. All are thought to cotransfer vertically and thus share a common genealogy. However, MORFFOs represent an exception in fern plastomes, exhibiting inconsistent genic positions and genealogies (Kim & Kim, 2018, 2020; Robison *et al*., 2018; Lehtonen & Cárdenas, 2019; Kim *et al*., 2023; Kuo *et al*., 2024). Due to their ambiguous homology and dynamic nature, MORFFOs were only discovered recently, in 2018, initially from fern plastomes (Kim & Kim, 2018; Robison *et al*., 2018). Nevertheless, they appear more widespread in fern plastomes than previously recognized. In this study, we identified and characterized novel MORFFOs, *morffo4* and *morffo5*, which are found only in Anemiaceae and sister families. In addition to various loci in fern plastomes, MORFFOs have also been detected in mitochondrial and nuclear genomes in ferns and other plant lineages (Kuo *et al*., 2024). Despite their prevalence, MORFFOs do not appear essential for plastid and plastome function as they are absent from some fern plastomes, including some *Anemia* plastomes studied here (Fig. 1; Table 1).

Our comprehensive plastome sequencing in a discrete fern lineage, *Anemia*, offers plastome maps along with a fine‐scaled phylogenetic framework to investigate genomic dynamics of MORFFOs in greater detail. First, we found that transposition and replication of these genes are likely independent of plastomes. In *Anemia*, MORFFOs have shifted their plastomic locations across lineages (Fig. 2). However, *Anemia* plastomes exhibit no structural changes that disrupt gene synteny, providing a potential opportunity for gene translocation. Thus, we also found no additional gene transpositions in plastomes. Previous MORFFO studies echo these findings, and genic positions of MORFFOs were usually inconsistent, while irrelevant to plastomic rearrangements (Robison *et al*., 2018; Kuo *et al*., 2024). These phenomena suggest the capabilities of MORFFOs to replicate and move alone without relying on their plastomes. Evidence from their inconsistent genealogies and elevated substitution rates further supports this idea. While MORFFOs remain functionally constrained as demonstrated here (Figs 3 and 4), they show exceptionally higher substitution rates than that of other plastid‐encoded CDS (Fig. 5) – even those from the SSC and LSC, which usually have higher substitution rates than the CDSs present in the IR (Li *et al*., 2016). This pattern is likely the result of faster replication rates (i.e. cumulating more mutations through duplication cycles per unit time) or increased error rates during replication (e.g. using a different polymerase). In either case, MORFFO replication is suggested to be unsynchronized with, and uncoupled from, plastome replication, and may occur outside of plastids. Second, we found that MORFFOs do not appear to be essential for maintaining plastid function or overall fitness. They are frequently pseudogenized or absent in *Anemia* plastomes (Figs 2, 7, 8). In one notable case, their presence in *A. phyllitidis* plastomes shows dynamic variation even within this species. One of the sequenced plastomes lacks MORFFOs entirely (GenBank accession no.: OM990738; data not shown), and another expresses apparently functional MORFFOs (Dataset S1). Taken together, MORFFOs more resemble selfish and mobile DNA elements, characterized by their dynamic and often unpredictable presence, whereas canonical plastome CDS primarily have essential roles in plastid function.

### HGT acquisition of MORFFOs and their shuttling among plant lineages

Plastomes of land plants are generally conserved in their genic composition and even tend to resist the invasion of foreign DNA (Filip & Skuza, 2021; Cauz‐Santos, 2025), in contrast to the mitogenome and nuclear genome, in which transfers of foreign genes are more common (Davis & Xi, 2015; Filip & Skuza, 2021). Unsurprisingly, relatively few studies have confirmed HGT or IGT in plastomes of land plants (Straub *et al*., 2013; Filip & Skuza, 2021; Cauz‐Santos, 2025). However, the increasing discovery of MORFFOs in fern plastomes is reshaping this understanding. The dynamic presence of MORFFOs suggests frequent HGT and IGT events (Kim & Kim, 2018; Kuo *et al*., 2024). Previous evidence has largely been based on comparative genomics, including a few plastomes within select lineages and sequence similarity searches that found BLAST hits in GenBank (Kim & Kim, 2018, 2020; Robison *et al*., 2018; Kim *et al*., 2023). Nonetheless, direct phylogenetic evidence clearly identifying such HGT or IGT events has been lacking. Kuo *et al*. (2024) analyzed plastome and mitogenome assemblies from 16 representatives of the fern family Ophioglossaceae. This first phylogenetic investigation of fern MORFFOs revealed a scattered distribution of Ophioglossaceae sequences and interlaced patterns of plastid and mitochondrial sequences in their gene trees, suggesting that frequent HGT and/or IGT events have shaped their evolution. Due to scarce sampling of closely related taxa, the evolutionary trajectory and directionality of these transfers, however, remain unclear for Ophioglossaceae and other fern lineages.

Leveraging dense plastome sampling within a specific fern lineage and broadly across ferns, our study focuses on MORFFOs from *Anemia*. Although the origin of these MORFFOs is still not fully proved, the strong genealogical conflicts (i.e. UFBS > 90) between the MORFFO and the plastome phylogeny (Figs 7 and 8) suggest that MORFFO genes are more likely to have been transferred into fern plastomes through HGT and even IGT. The most striking example is *A. simii*, whose MORFFOs, *morffo1* to *morffo3*, are phylogenetically nested within plastid sequences from distantly related fern lineages, mostly from Polypodiales suborder Pteridineae (Figs 7, S3–S5). This opens up the possibility of HGT from Pteridineae (Polypodiales) and/or other distantly related fern lineages into *A. simii*, although additional evidence is needed to ascertain whether the transfer originated directly from the plastome or from other genomic compartments (i.e. mitogenome or nuclear genome) in these fern lineages. It also remains possible that some of the MORFFOs in *A. simii* and those in Pteridineae plastomes share a common origin in nonplastid genomes, subsequently being inserted into plastomes through HGT and IGT. Additional evidence supporting this HGT hypothesis is that the *A. simii* plastome lacks *morffo4* and *morffo5*, which are otherwise unique to Schizaeales plastomes (Fig. 2). Another notable example of HGT involves *Anemia morffo1* and *morffo2*, which appear to have been transferred into the plastomes of distantly related fern lineages, including *O. gibba* (Aspleniineae; Polypodiales) and *D. truncatula* (Polypodiineae; Polypodiales) (Fig. 7). In these recipient plastomes, *Anemia*‐like MORFFOs are inserted at different positions, and arranged differently from the configuration in *Anemia* (Fig. S6). Even more intriguing, in the plastome of *O. gibba*, the *Anemia*‐like MORFFOs are flanked by *morffo3* genes apparently related to the fern family Desmophlebiaceae, which are likely to have originated from a distinct HGT source (Fig. S5). These findings indicate that MORFFO genes not only can translocate independently but also may form chimeric gene blocks that insert together into a plastome.

Although the presence of MORFFOs in the mitogenome and nuclear genome of *Anemia* remains understudied due to the difficulty of fully assembling these genomes, their presence is highly plausible, as suggested by Kuo *et al*. (2024). Through BLAST searches in the *A. phyllitidis* transcriptome, we identified sequence‐variant MORFFO copies that are absent from its plastome. These findings support their origin outside of plastomes, likely from the nuclear genome, as indicated by their RSCU similarity (Fig. 6b). Interestingly, the absence of these copies in poly‐A–enriched transcriptomes suggests that they are not typical nuclear mRNAs. Instead, they may represent viral‐like/selfish genetic elements residing in the nuclear genome. Our preliminary search of the nuclear genome of its sister genus *Lygodium* (Pelosi *et al*., 2025) also identified sequences closely matching MORFFOs (data not shown). Importantly, our deep investigation of MORFFO evolutionary patterns in *Anemia* demonstrates that these genes are likely transcribed, translated, and replicated outside the plastids, supporting their mechanistic potential to facilitate IGT‐based transfer.

### Conclusions

MORFFOs in fern plastomes remain largely mysterious. The molecular mechanisms underlying their replication and insertion into these genomes are still unknown, despite their widespread presence across distinct fern lineages. In this study, we identified two novel MORFFOs from the fern family Anemiaceae. Our comparative genomic and phylogenetic approaches further reveal their evolutionary constraints as CDS, yet also their remarkable evolutionary mobility, contrasting with the steady genomic ‘plate’ that is the rest of the plastome, in which gene synteny has been maintained for more than 150 Myr, as showcased in the ancient fern lineage Anemiaceae. MORFFOs also display unusual genetic signatures that differ from plastid‐encoded CDS, including the absence of RNA editing, distinct codon usage, and extraordinarily high substitution rates. These diagnostics suggest their possible origins outside of the plastome, potentially involving HGT and/or IGT. Notably, these findings underscore that such mobile elements can move across genic, genomic, and even interspecies barriers, highlighting unique cases in which fern plastomes have frequently acquired genes through HGT or IGT, a phenomenon rarely observed in other land plant lineages.

## Competing interests

None declared.

## Author contributions

PHL was involved in conceptualization, data curation, analysis, funding acquisition, investigation, methodology, writing – original draft, writing – review and editing. L.‐YK was involved in conceptualization, data curation, analysis, investigation, methodology, writing – original draft, writing – review and editing. BDF was involved in analysis, methodology, writing – original draft, writing – review and editing. KGK was involved in conceptualization, data curation, analysis, funding acquisition, investigation, methodology, writing – original draft, writing – review and editing.

## Disclaimer

The New Phytologist Foundation remains neutral with regard to jurisdictional claims in maps and in any institutional affiliations.

## Supporting information


**Dataset S1** Voucher information and the results of the BLAST similarity searches of the *Anemia* MORFFOs.

**Dataset S2** Plastid RNA editing sites for *Anemia phyllitidis*.

**Fig. S1** Phylogram of the Schizaeales based on 86 plastid CDS.
**Fig. S2** Relative and absolute substitution rates of *Anemia* MORFFOs and the plastid CDSs.
**Fig. S3** Phylogeny of *morffo1*.
**Fig. S4** Phylogeny of *morffo2*.
**Fig. S5** Phylogeny of *morffo3*.
**Fig. S6** Organization of *morffo* genes in the plastome of *Oceaniopteris gibba*.
Please note: Wiley is not responsible for the content or functionality of any Supporting Information supplied by the authors. Any queries (other than missing material) should be directed to the *New Phytologist* Central Office.

## Data Availability

Illumina reads of genome skimming and the transcriptome are submitted to the Sequence Read Archive (SRA) under BioProject accession nos. PRJNA1285353 and PRJNA1291224, respectively. The assembled plastome sequences are deposited in NCBI under accession nos. PV764656–PV764711 and PV938982, and their gff files with the annotation are provided at https://github.com/lykuofern/Anemia.
