# Supplementary material for: Evolutionary mobility and genetic dynamics of MORFFO genes: shuttling among ancient plant lineages
Source: New Phytol. 2026 Feb 19;250(2):1248–64. doi: 10.1111/nph.70986 (PMC13000998; doi:10.1111/nph.70986)
Supplement: Supplementary file 3 — Fig. S1 Phylogram of the Schizaeales based on 86 plastid CDS. Fig. S2 Relative and absolute substitution rates of Anemia MORFFOs and the plastid CDSs. Fig. S3 Phylogeny of morffo1. Fig. S4 Phylogeny of morffo2. Fig. S5 Phylogeny of morffo3. Fig. S6 Organization of morffo genes in the plastome of Oceaniopteris gibba. Please note: Wiley is not responsible for the content or functionality of any Supporting Information supplied by the authors. Any queries (other than missing material) should be directed to the New Phytologist Central Office. [file NPH-250-1248-s001.pdf]

New Phytologist Supporting Information

Article title: Evolutionary mobility and genetic dynamics of MORFFO genes: shuttling among ancient plant lineages

Authors: Paulo H. Labiak, Li-Yaung Kuo, Blake D. Fauskee, Kenneth G. Karol

Article acceptance date: 18 January 2026

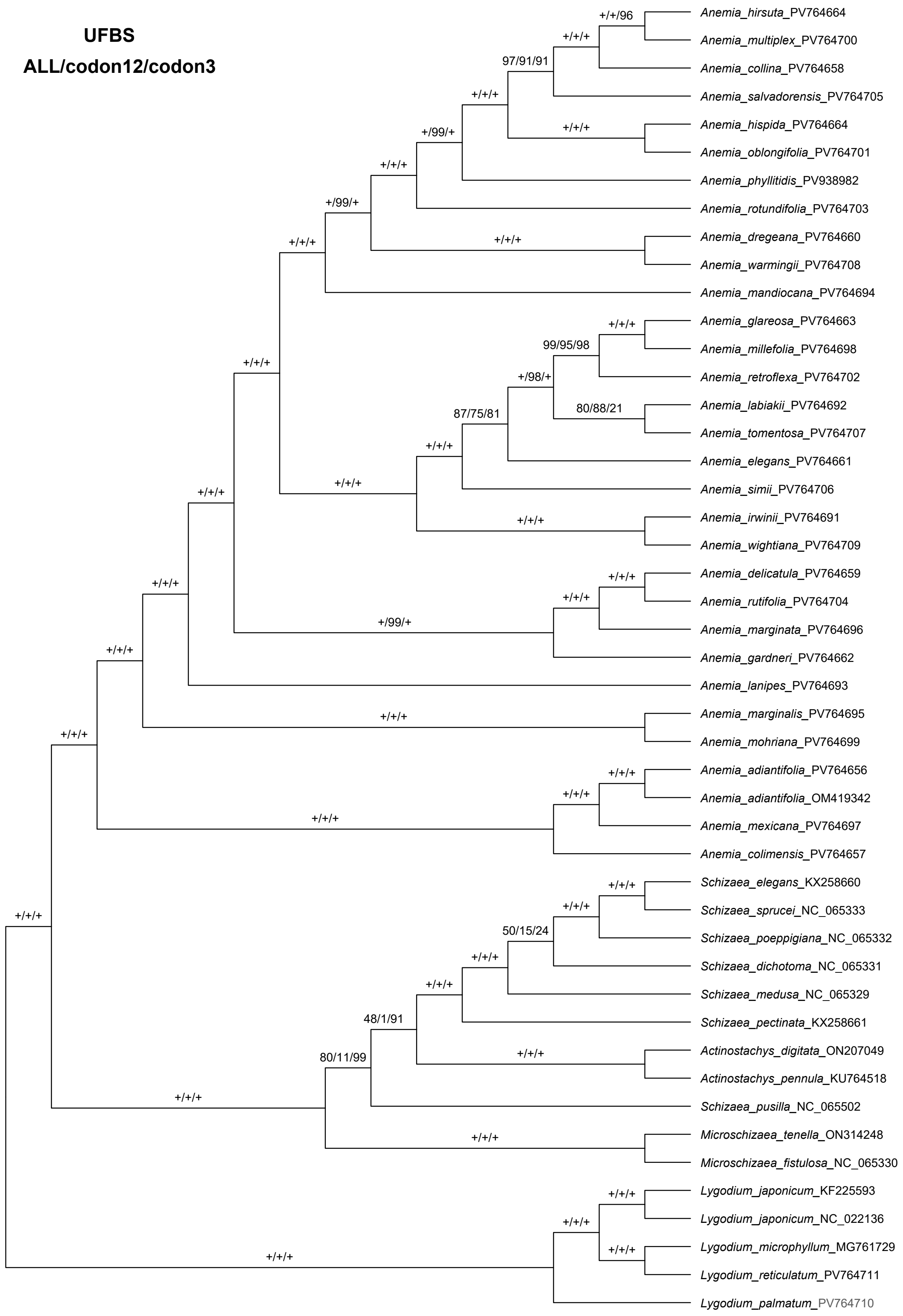

Fig. S1. A phylogram of the Schizaeales based on 86 plastid CDS. Values shown along branches are ML ultrafast bootstrap supports (UFBS) inferred from three codon partitioning schemes (i.e., "ALL", "codon12", "codon3"); '+' indicates a value of 100.

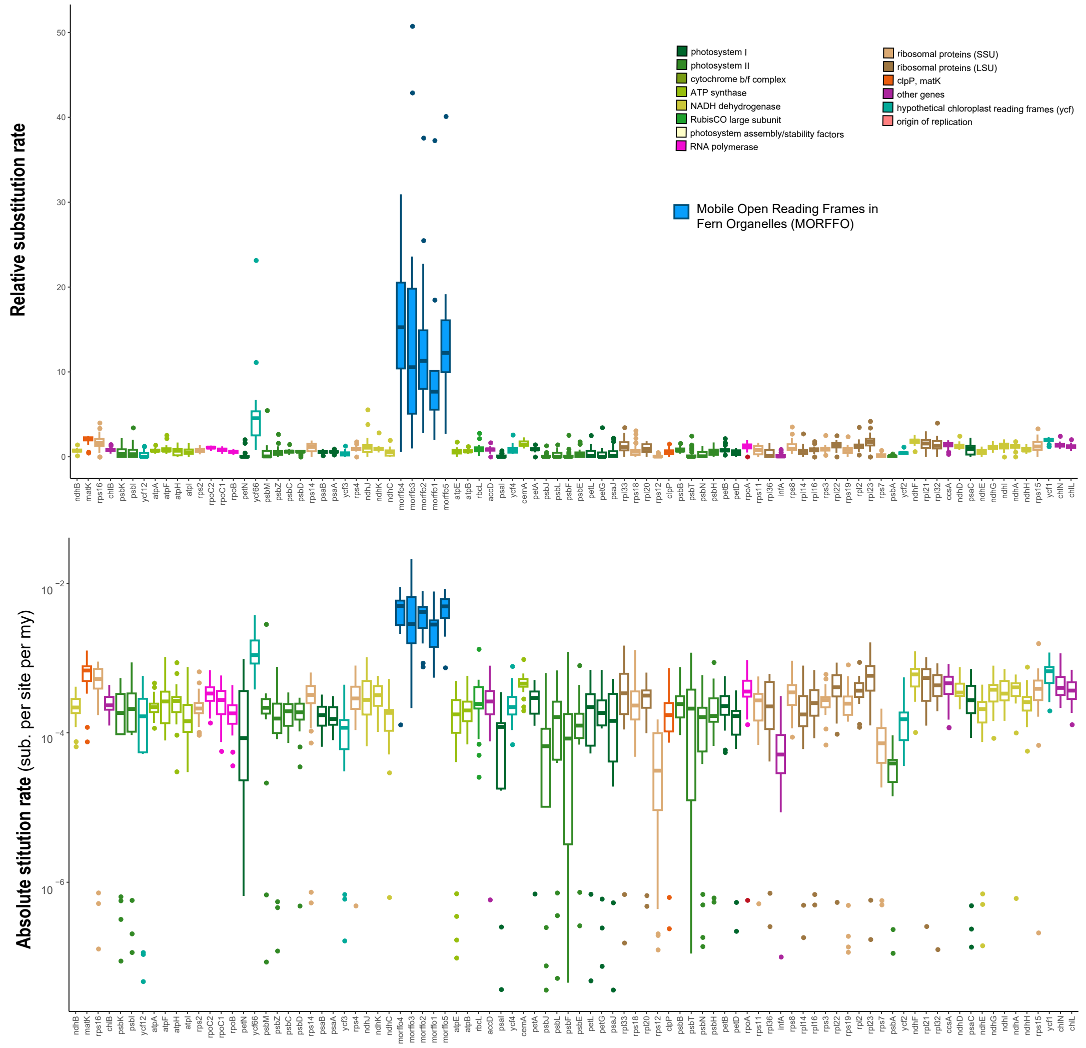

Fig. S2. Relative (upper) and absolute (lower) substitution rates of MORFFOs, including pseudogene-like sequences, and 86 plastid CDS in *Anemia*.

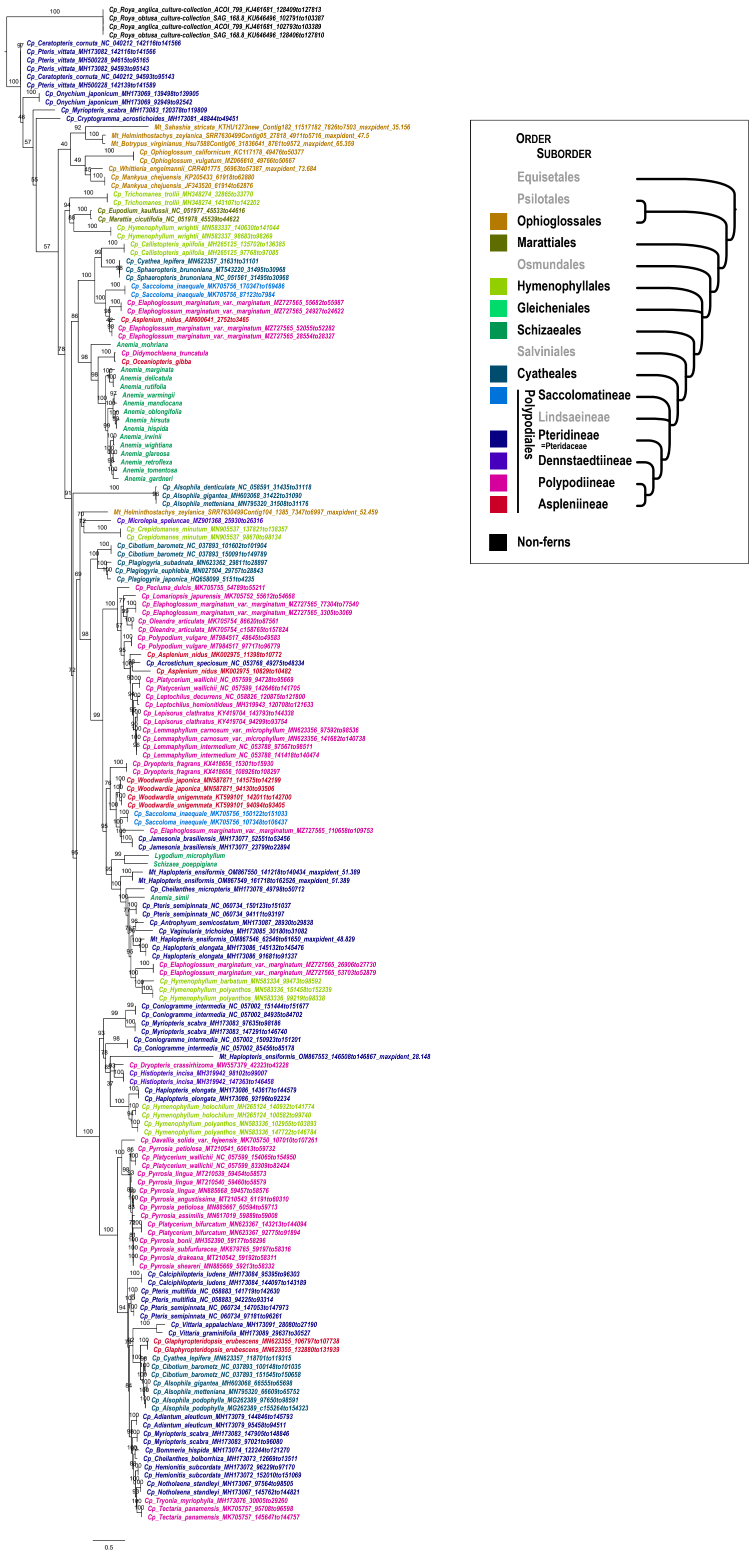

Fig. S3. Phylogeny of *morffo1*. Each tip name includes the following information: genomic origin (Cp = plant plastid; Mt = mitochondrion), GeneBank accession number, and site positions. For the plastomic ones, the genic position is indicated in bold behind their tip names. The values on the branches are ML UFBS.

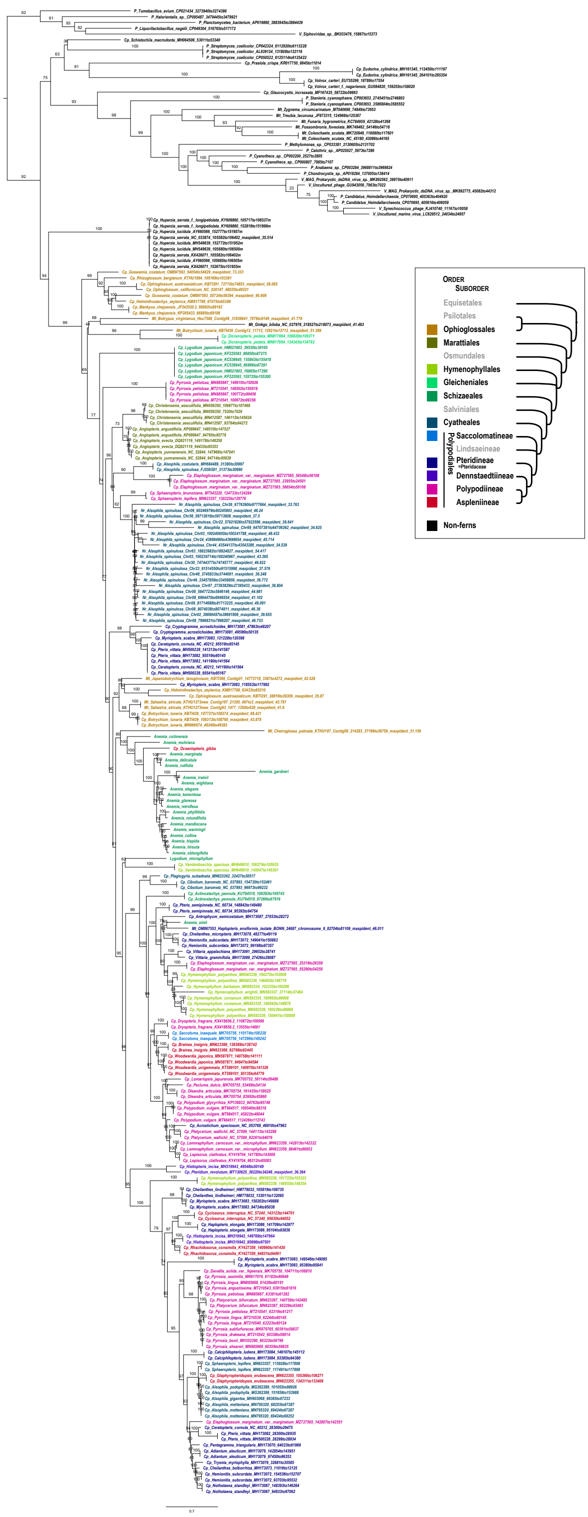

Fig. S4. Phylogeny of *morffo2*. Each tip name includes the following information: genomic origin (Cp = plant plastid, Mt = mitochondrion, Nr = nuclear, P = prokaryotic plasmid, V = virus or phage), GeneBank accession number, and site positions. For the plastomic ones, the genic position is indicated in bold behind their tip names. The values on the branches are ML UFBS.

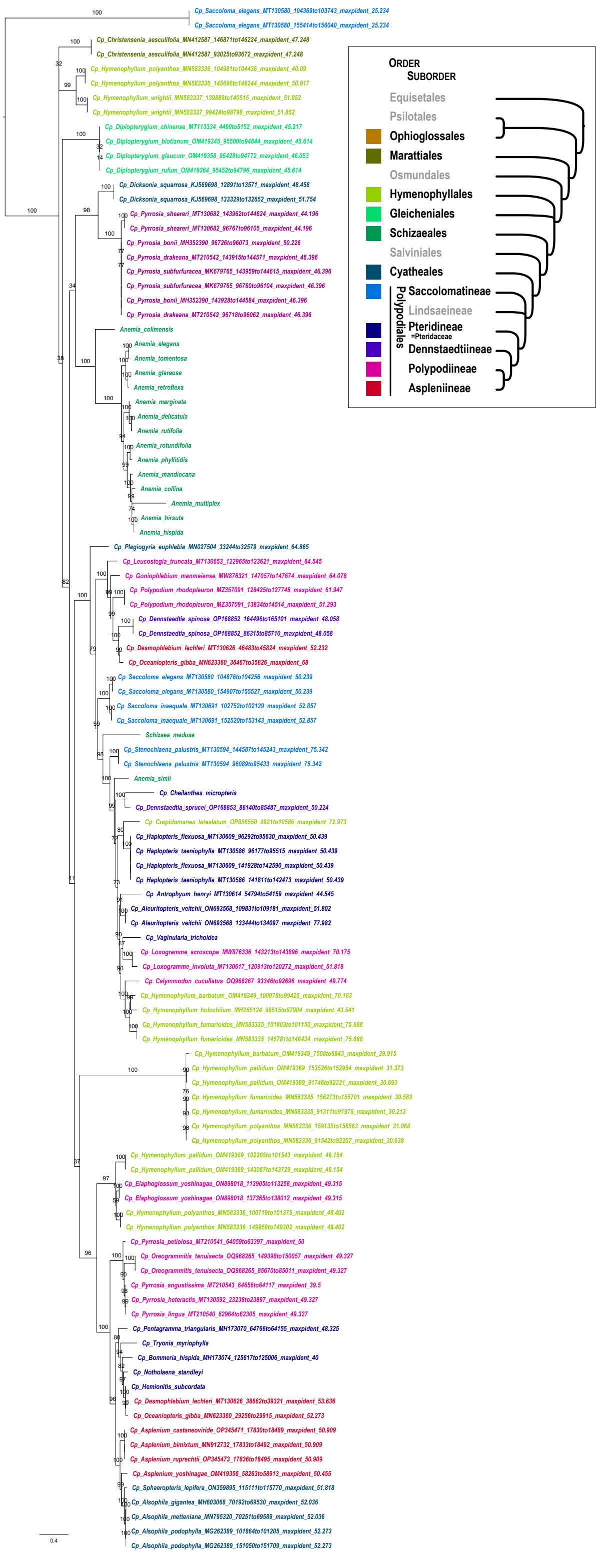

Fig. S5. Phylogeny of *morffo3*. Each tip name includes the following information: genomic origin (Cp = plant plastid), GeneBank accession number, and site positions. For the plastomic ones, the genic position is indicated in bold behind their tip names. The values on the branches are ML UFBS.

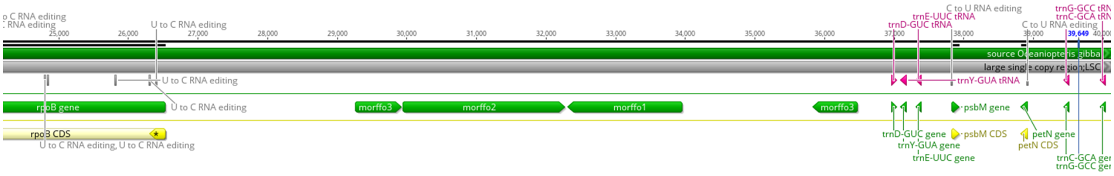

Fig. S6. Organization of *morffo* genes in the plastome of *Oceaniopteris gibba* (GenBank accession: MN623360).
